# 3-[(2-Chloro-1,3-thia­zol-5-yl)meth­yl]-5-methyl-1,3,5-oxadiazinan-4-one

**DOI:** 10.1107/S1600536812042031

**Published:** 2012-10-13

**Authors:** Rajni Kant, Vivek K. Gupta, Kamini Kapoor, Chetan S. Shripanavar, Kaushik Banerjee

**Affiliations:** a X-ray Crystallography Laboratory, Post-Graduate Department of Physics & Electronics, University of Jammu, Jammu Tawi 180 006, India; b National Research Centre for Grapes, Pune 412 307, India

## Abstract

In the title compound, C_8_H_10_ClN_3_O_2_S, the oxadiazinane ring is in a sofa conformation with the ring O atom deviating from the best plane of the remaining five atoms by 0.636 (2) Å. A short intra­molecular C-S⋯O=C contact [S⋯O 3.122 (2) Å, C—S⋯O 80.0 (2)°] is observed between the two mol­ecular fragments bridged by the methyl­ene group. In the crystal, C—H⋯O hydrogen bonds link mol­ecules, forming chains along the *b* axis.

## Related literature

For the biological activity of thia­methoxam, see: Maienfisch *et al.* (2001 ▶, 2006 ▶); Suchail *et al.* (2001 ▶); Ford & Casida (2006 ▶). For the structure of thia­methoxam, see: Chopra *et al.* (2004 ▶). For ring conformations, see: Duax & Norton (1975 ▶).
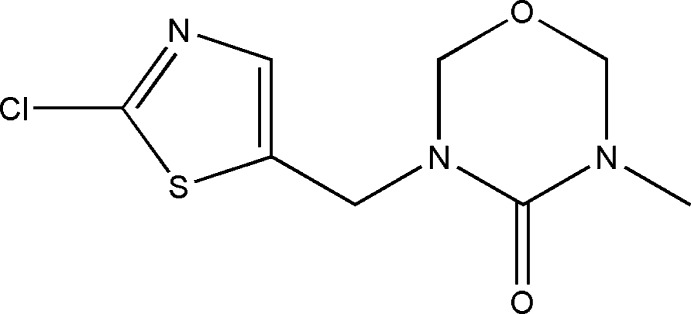


## Experimental

### 

#### Crystal data

C_8_H_10_ClN_3_O_2_S
*M* *_r_* = 247.70
Orthorhombic, 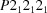

*a* = 4.6141 (2) Å
*b* = 11.7335 (4) Å
*c* = 20.1460 (8) Å
*V* = 1090.70 (7) Å^3^
*Z* = 4
Mo *K*α radiation
μ = 0.53 mm^−1^
*T* = 293 K
0.3 × 0.2 × 0.2 mm

#### Data collection

Oxford Diffraction Xcalibur Sapphire3 diffractometer
Absorption correction: multi-scan (*CrysAlis PRO*; Oxford Diffraction, 2010 ▶) *T*_min_ = 0.925, *T*_max_ = 1.000
22323 measured reflections
2147 independent reflections
1974 reflections with *I* > 2σ(*I*)
*R*_int_ = 0.034

#### Refinement

*R*[*F* ^2^ > 2σ(*F* ^2^)] = 0.031
*wR*(*F* ^2^) = 0.081
*S* = 1.07
2147 reflections
137 parameters
H-atom parameters constrained
Δρ_max_ = 0.22 e Å^−3^
Δρ_min_ = −0.16 e Å^−3^
Absolute structure: Flack (1983 ▶), 856 Friedel pairs
Flack parameter: 0.04 (9)

### 

Data collection: *CrysAlis PRO* (Oxford Diffraction, 2010 ▶); cell refinement: *CrysAlis PRO*; data reduction: *CrysAlis PRO*; program(s) used to solve structure: *SHELXS97* (Sheldrick, 2008 ▶); program(s) used to refine structure: *SHELXL97* (Sheldrick, 2008 ▶); molecular graphics: *ORTEP-3* (Farrugia, 1997 ▶); software used to prepare material for publication: *PLATON* (Spek, 2009 ▶).

## Supplementary Material

Crystal structure: contains datablock(s) I, New_Global_Publ_Block. DOI: 10.1107/S1600536812042031/gk2519sup1.cif

Structure factors: contains datablock(s) I. DOI: 10.1107/S1600536812042031/gk2519Isup2.hkl

Supplementary material file. DOI: 10.1107/S1600536812042031/gk2519Isup3.cml

Additional supplementary materials:  crystallographic information; 3D view; checkCIF report

## Figures and Tables

**Table 1:** Hydrogen-bond geometry (Å, °)

*D*—H⋯*A*	*D*—H	H⋯*A*	*D*⋯*A*	*D*—H⋯*A*
C12—H12⋯O7^i^	0.93	2.60	3.443 (3)	151

Symmetry code: (i) 
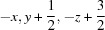
.

**Fig. 1.**
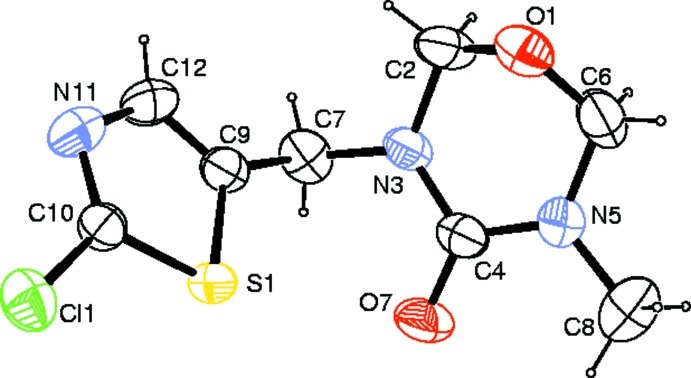
ORTEP view of the molecule with the atom-labeling scheme. The thermal ellipsoids are drawn at the 40% probability level. H atoms are shown as small spheres of arbitrary radii.

**Fig. 2.**
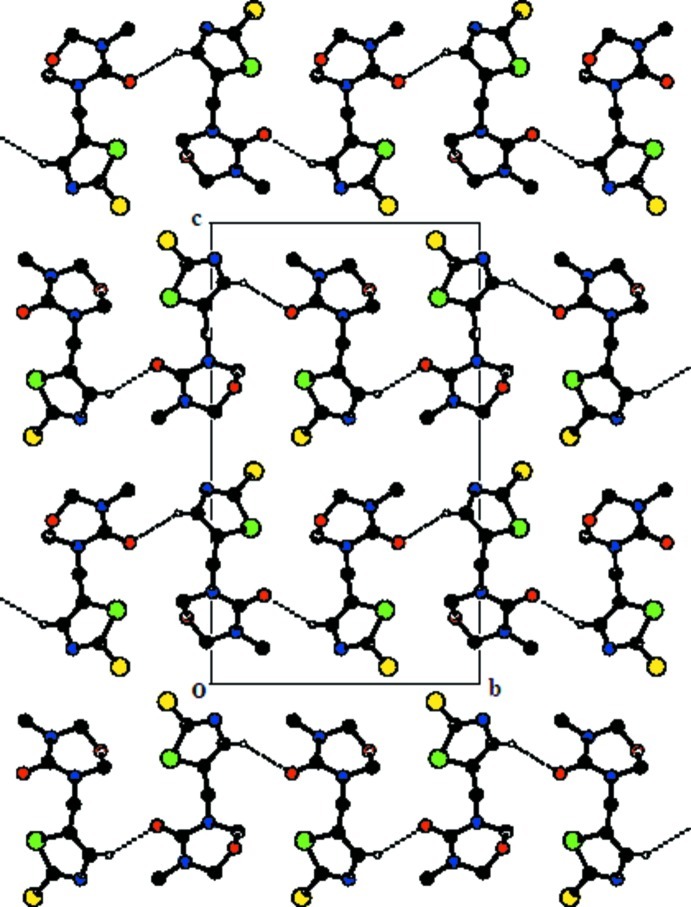
The packing arrangement of molecules viewed down the a axis. The dotted lines show intermolecular C—H···O hydrogen bonds.

## supplementary crystallographic information

### Comment

An important milestone in the history of modern insect control is marked by the discovery of neonicotinoid insecticides (Maienfisch, 2006). In 1998 Novartis launched thiamethoxam as a novel second generation neonicotinoid with a unique structure and outstanding insecticidal activity (Maienfisch *et al.*, 2001). The major natural metabolite of thiamethoxam is the title compound, which is thiamethoxam urea derivative (Suchail *et al.*, 2001, Ford & Casida, 2006)

In the title compound (Fig.1) all bond lengths and angles are normal and correspond to those observed in the related structure (Chopra *et al.*, 2004). The oxadiazinane ring is in a sofa conformation [asymmetry parameter: ΔCs(O1—C4) = 7.47 (Duax & Norton, 1975)]. In the crystal, the displacement of the atom O1 from the plane defined by atoms C2/N3/C4/N5/C6 is -0.636 (2) Å. In thiametoxam and the title compound the two molecular fragments bridged by the methylene group are similarly oriented. C—H···O hydrogen bonds link molecules to form chains along *b* axis(Fig.2).

### Experimental

Thiamethoxam (0.291 g, 0.001 mol) was dissolved in 5 ml methanol and to it 5 ml of 1 N K_2_CO_3_ solution was added. The reaction mixture was refluxed for about 10 h on a water bath at 343 K and then cooled. The reaction mixture was neutralized with 1 N HCl solution, until the solid compound was separated out. The synthesized compound was dissolved in minimum amount of methanol and was kept standing for slow evaporation until colourless transparent crystals were formed (m.p. = 372 K).

### Refinement

All H atoms were positioned geometrically and were treated as riding on their parent C atoms, with C—H distances of 0.93–0.97 Å and with *U*_iso_(H) = 1.2*U*_eq_(C) or 1.5*U*_eq_(methyl C).

### Crystal data

**Table:**

C_8_H_10_ClN_3_O_2_S	*F*(000) = 512
*M**_r_* = 247.70	*D*_x_ = 1.508 Mg m^−^^3^
Orthorhombic, *P*2_1_2_1_2_1_	Mo *K*α radiation, λ = 0.71073 Å
Hall symbol: P 2ac 2ab	Cell parameters from 11280 reflections
*a* = 4.6141 (2) Å	θ = 3.5–29.0°
*b* = 11.7335 (4) Å	µ = 0.53 mm^−^^1^
*c* = 20.1460 (8) Å	*T* = 293 K
*V* = 1090.70 (7) Å^3^	Needle, white
*Z* = 4	0.3 × 0.2 × 0.2 mm

### Data collection

**Table:**

Oxford Diffraction Xcalibur Sapphire3 diffractometer	2147 independent reflections
Radiation source: fine-focus sealed tube	1974 reflections with *I* > 2σ(*I*)
Graphite monochromator	*R*_int_ = 0.034
Detector resolution: 16.1049 pixels mm^-1^	θ_max_ = 26.0°, θ_min_ = 3.5°
ω scan	*h* = −5→5
Absorption correction: multi-scan (*CrysAlis PRO*; Oxford Diffraction, 2010)	*k* = −14→14
*T*_min_ = 0.925, *T*_max_ = 1.000	*l* = −24→24
22323 measured reflections	

### Refinement

**Table:**

Refinement on *F*^2^	Secondary atom site location: difference Fourier map
Least-squares matrix: full	Hydrogen site location: inferred from neighbouring sites
*R*[*F*^2^ > 2σ(*F*^2^)] = 0.031	H-atom parameters constrained
*wR*(*F*^2^) = 0.081	*w* = 1/[σ^2^(*F*_o_^2^) + (0.0399*P*)^2^ + 0.2799*P*] where *P* = (*F*_o_^2^ + 2*F*_c_^2^)/3
*S* = 1.07	(Δ/σ)_max_ = 0.001
2147 reflections	Δρ_max_ = 0.22 e Å^−^^3^
137 parameters	Δρ_min_ = −0.16 e Å^−^^3^
0 restraints	Absolute structure: Flack (1983), 856 Friedel pairs
Primary atom site location: structure-invariant direct methods	Flack parameter: 0.04 (9)

### Special details

**Table:**

Experimental. *CrysAlis PRO*, Oxford Diffraction Ltd., Version 1.171.34.40 (release 27–08-2010 CrysAlis171. NET) (compiled Aug 27 2010,11:50:40) Empirical absorption correction using spherical harmonics, implemented in SCALE3 ABSPACK scaling algorithm.
Geometry. All e.s.d.'s (except the e.s.d. in the dihedral angle between two l.s. planes) are estimated using the full covariance matrix. The cell e.s.d.'s are taken into account individually in the estimation of e.s.d.'s in distances, angles and torsion angles; correlations between e.s.d.'s in cell parameters are only used when they are defined by crystal symmetry. An approximate (isotropic) treatment of cell e.s.d.'s is used for estimating e.s.d.'s involving l.s. planes.
Refinement. Refinement of *F*^2^ against ALL reflections. The weighted *R*-factor *wR* and goodness of fit *S* are based on *F*^2^, conventional *R*-factors *R* are based on *F*, with *F* set to zero for negative *F*^2^. The threshold expression of *F*^2^ > σ(*F*^2^) is used only for calculating *R*-factors(gt) *etc*. and is not relevant to the choice of reflections for refinement. *R*-factors based on *F*^2^ are statistically about twice as large as those based on *F*, and *R*- factors based on ALL data will be even larger.

### Fractional atomic coordinates and isotropic or equivalent isotropic displacement parameters (Å^2^)

**Table:**

	*x*	*y*	*z*	*U*_iso_*/*U*_eq_	
S1	0.29172 (13)	0.85031 (5)	0.83926 (3)	0.04611 (15)	
Cl1	0.64157 (15)	0.83860 (6)	0.96232 (3)	0.0642 (2)	
O1	0.4809 (4)	1.08899 (16)	0.64448 (10)	0.0653 (5)	
C2	0.2262 (7)	1.1021 (2)	0.68003 (16)	0.0668 (8)	
H2A	0.0844	1.1412	0.6527	0.080*	
H2B	0.2630	1.1487	0.7189	0.080*	
N3	0.1108 (5)	0.99332 (17)	0.70052 (10)	0.0512 (5)	
C4	0.1836 (6)	0.8940 (2)	0.67011 (12)	0.0516 (6)	
N5	0.3433 (6)	0.90503 (19)	0.61443 (11)	0.0640 (6)	
C6	0.4364 (9)	1.0158 (3)	0.59173 (14)	0.0778 (9)	
H6A	0.6149	1.0079	0.5667	0.093*	
H6B	0.2905	1.0475	0.5624	0.093*	
C7	−0.0657 (6)	0.9905 (3)	0.76014 (14)	0.0613 (7)	
H7A	−0.2048	0.9289	0.7564	0.074*	
H7B	−0.1734	1.0613	0.7634	0.074*	
O7	0.1066 (5)	0.80128 (15)	0.69297 (10)	0.0744 (6)	
C8	0.4470 (9)	0.8075 (3)	0.57872 (19)	0.0975 (12)	
H8A	0.3872	0.7392	0.6012	0.146*	
H8B	0.6547	0.8098	0.5765	0.146*	
H8C	0.3684	0.8080	0.5346	0.146*	
C9	0.1052 (5)	0.9747 (2)	0.82176 (12)	0.0494 (6)	
C10	0.4123 (5)	0.9127 (2)	0.91085 (12)	0.0484 (6)	
N11	0.3276 (6)	1.01443 (19)	0.92255 (12)	0.0692 (7)	
C12	0.1516 (7)	1.0488 (2)	0.87096 (15)	0.0680 (8)	
H12	0.0683	1.1210	0.8704	0.082*	

### Atomic displacement parameters (Å^2^)

**Table:**

	*U*^11^	*U*^22^	*U*^33^	*U*^12^	*U*^13^	*U*^23^
S1	0.0529 (3)	0.0353 (2)	0.0501 (3)	0.0000 (2)	0.0022 (2)	0.0000 (2)
Cl1	0.0651 (4)	0.0728 (4)	0.0546 (4)	−0.0027 (4)	−0.0063 (3)	0.0091 (3)
O1	0.0633 (11)	0.0579 (11)	0.0748 (12)	−0.0128 (9)	−0.0003 (10)	0.0120 (10)
C2	0.0732 (18)	0.0395 (12)	0.088 (2)	0.0011 (13)	0.0047 (16)	0.0129 (13)
N3	0.0552 (12)	0.0421 (10)	0.0563 (12)	−0.0002 (9)	0.0042 (10)	0.0105 (9)
C4	0.0570 (13)	0.0446 (11)	0.0533 (14)	−0.0083 (11)	−0.0123 (13)	0.0084 (11)
N5	0.0871 (17)	0.0551 (13)	0.0499 (12)	−0.0056 (13)	0.0064 (12)	−0.0020 (10)
C6	0.102 (3)	0.079 (2)	0.0529 (17)	−0.0165 (19)	0.0084 (16)	0.0135 (15)
C7	0.0480 (14)	0.0691 (16)	0.0669 (17)	0.0082 (13)	0.0019 (12)	0.0110 (14)
O7	0.1021 (17)	0.0435 (9)	0.0775 (13)	−0.0221 (11)	−0.0017 (13)	0.0082 (9)
C8	0.121 (3)	0.089 (2)	0.083 (2)	0.003 (2)	0.011 (2)	−0.025 (2)
C9	0.0472 (13)	0.0450 (12)	0.0561 (14)	0.0058 (10)	0.0112 (11)	0.0061 (11)
C10	0.0505 (13)	0.0459 (13)	0.0487 (13)	−0.0061 (11)	0.0058 (11)	0.0009 (10)
N11	0.0892 (19)	0.0501 (12)	0.0683 (15)	0.0053 (13)	0.0002 (14)	−0.0144 (11)
C12	0.087 (2)	0.0429 (14)	0.0741 (19)	0.0176 (14)	0.0026 (17)	−0.0061 (12)

### Geometric parameters (Å, º)

**Table:**

S1—C10	1.710 (2)	N5—C6	1.443 (4)
S1—C9	1.731 (2)	C6—H6A	0.9700
Cl1—C10	1.718 (3)	C6—H6B	0.9700
O1—C6	1.382 (4)	C7—C9	1.482 (4)
O1—C2	1.385 (4)	C7—H7A	0.9700
C2—N3	1.443 (3)	C7—H7B	0.9700
C2—H2A	0.9700	C8—H8A	0.9600
C2—H2B	0.9700	C8—H8B	0.9600
N3—C4	1.359 (3)	C8—H8C	0.9600
N3—C7	1.452 (3)	C9—C12	1.336 (4)
C4—O7	1.233 (3)	C10—N11	1.278 (3)
C4—N5	1.348 (3)	N11—C12	1.379 (4)
N5—C8	1.434 (4)	C12—H12	0.9300
			
C10—S1—C9	88.42 (12)	N3—C7—C9	113.4 (2)
C6—O1—C2	109.9 (2)	N3—C7—H7A	108.9
O1—C2—N3	111.3 (2)	C9—C7—H7A	108.9
O1—C2—H2A	109.4	N3—C7—H7B	108.9
N3—C2—H2A	109.4	C9—C7—H7B	108.9
O1—C2—H2B	109.4	H7A—C7—H7B	107.7
N3—C2—H2B	109.4	N5—C8—H8A	109.5
H2A—C2—H2B	108.0	N5—C8—H8B	109.5
C4—N3—C2	122.6 (2)	H8A—C8—H8B	109.5
C4—N3—C7	119.5 (2)	N5—C8—H8C	109.5
C2—N3—C7	117.6 (2)	H8A—C8—H8C	109.5
O7—C4—N5	123.6 (2)	H8B—C8—H8C	109.5
O7—C4—N3	121.1 (2)	C12—C9—C7	128.7 (2)
N5—C4—N3	115.3 (2)	C12—C9—S1	108.5 (2)
C4—N5—C8	121.5 (3)	C7—C9—S1	122.7 (2)
C4—N5—C6	120.9 (2)	N11—C10—S1	117.1 (2)
C8—N5—C6	117.4 (3)	N11—C10—Cl1	123.4 (2)
O1—C6—N5	111.1 (2)	S1—C10—Cl1	119.52 (14)
O1—C6—H6A	109.4	C10—N11—C12	108.3 (2)
N5—C6—H6A	109.4	C9—C12—N11	117.6 (2)
O1—C6—H6B	109.4	C9—C12—H12	121.2
N5—C6—H6B	109.4	N11—C12—H12	121.2
H6A—C6—H6B	108.0		
			
C6—O1—C2—N3	54.5 (3)	C4—N3—C7—C9	85.9 (3)
O1—C2—N3—C4	−20.9 (4)	C2—N3—C7—C9	−87.9 (3)
O1—C2—N3—C7	152.7 (2)	N3—C7—C9—C12	111.4 (3)
C2—N3—C4—O7	172.1 (3)	N3—C7—C9—S1	−66.6 (3)
C7—N3—C4—O7	−1.3 (4)	C10—S1—C9—C12	−0.2 (2)
C2—N3—C4—N5	−7.7 (4)	C10—S1—C9—C7	178.1 (2)
C7—N3—C4—N5	178.8 (2)	C9—S1—C10—N11	0.3 (2)
O7—C4—N5—C8	−2.7 (5)	C9—S1—C10—Cl1	−178.75 (16)
N3—C4—N5—C8	177.2 (3)	S1—C10—N11—C12	−0.2 (3)
O7—C4—N5—C6	−177.5 (3)	Cl1—C10—N11—C12	178.8 (2)
N3—C4—N5—C6	2.4 (4)	C7—C9—C12—N11	−178.0 (3)
C2—O1—C6—N5	−59.9 (4)	S1—C9—C12—N11	0.2 (4)
C4—N5—C6—O1	31.4 (4)	C10—N11—C12—C9	0.0 (4)
C8—N5—C6—O1	−143.6 (3)		

### Hydrogen-bond geometry (Å, º)

**Table:**

*D*—H···*A*	*D*—H	H···*A*	*D*···*A*	*D*—H···*A*
C12—H12···O7^i^	0.93	2.60	3.443 (3)	151

Symmetry code: (i) −*x*, *y*+1/2, −*z*+3/2.
